# Microstructure Formation of Functional Polymers by Evaporative Self-Assembly under Flexible Geometric Confinement

**DOI:** 10.3390/mi9030124

**Published:** 2018-03-12

**Authors:** Xiangmeng Li, Xijing Zhu, Huifen Wei

**Affiliations:** 1 Shanxi Province Key Laboratory of Advanced Manufacturing Technology, North University of China, Taiyuan 030051, Shanxi, China; zxj161501@nuc.edu.cn; 2 Institute of Precision & Special Manufacturing, School of Mechanical Engineering, North University of China, Taiyuan 030051, Shanxi, China; 3 Academy of Science and Technology, North University of China, Taiyuan 030051, Shanxi, China; whf_nuc@163.com

**Keywords:** polymer, microstructure, nanocrystalline, flexible geometric confinement, evaporative self-assembly, field-effect transistor

## Abstract

Polymer microstructures are widely used in optics, flexible electronics, and so forth. We demonstrate a cost-effective bottom-up manner for patterning polymer microstructures by evaporative self-assembly under a flexible geometric confinement at a high temperature. Two-parallel-plates confinement would become curve-to-flat shape geometric confinement as the polydimethylsiloxane (PDMS) cover plate deformed during solvent swelling. We found that a flexible cover plate would be favorable for the formation of gradient microstructures, with various periodicities and widths obtained at varied heights of clearance. After thermal annealing, the edge of the PMMA (Poly-methylmethacrylate) microstructures would become smooth, while the RR-P3HT (regioregular-poly(3-hexylthiophene)) might generate nanocrystals. The morphologies of RR-P3HT structures included thick films, straight lines, hierarchical stripes, incomplete stripes, and regular dots. Finally, a simple field-effect transistor (FET) device was demonstrated with the RR-P3HT micropattern as an active layer.

## 1. Introduction

Evaporative self-assembly under geometric confinement is a simple bottom-up facial manner for fabricating microstructures of various nanomaterials which are widely used in applications of optics, optoelectronics, flexible electronics, bioengineering, and so forth [1,2,3,4,5,6,7,8,9]. In recent years, functional polymers have been patterned into highly ordered microstructures using many kinds of geometric confinement. As reported by Lin et al., stable and fix shapes of geometric confinement including two parallel plates, curve-to-flat and wedge-to-flat confinement, were able to form regular stripes in large area [1,2,3,4,10,11,12]. To the best of our knowledge, most of the abovementioned studies have reported on the self-assembly under a rigid geometric confinement rather than a flexible one. It is easy to figure out that the opening of the geometric confinement would influence the evaporative rate. Hence, it is desirable for the opening of the geometric confinement to be varied in an easy manner. Moreover, it would take long time to produce a polymer microstructure at low temperature due to the low evaporation rate, while a high temperature could enhance the evaporative induced self-assembly process of patterning polymeric microstructures. In addition, we have proposed in previous work that low temperatures were not favorable for the regular stripe formation of nanoparticles as well as for other nanomaterials [13]. Therefore, the effect of high temperatures in evaporative self-assembly should be further studied, especially for some solvents with high boiling points, in order that the formation of microstructure can be promoted.

Poly-methylmethacrylate (PMMA) as a kind of structural polymer, and regioregular-poly(3-hexylthiophene) (RR-P3HT) as a functional polymer with semiconducting property, were commonly used [9,14,15,16,17,18]. For instance, PMMA gratings could be used as diffractive components for optical devices or as a template for replicating other functional patterns [11,19]. The latter one, RR-P3HT, is widely used in organic solar cells and often used as an electron donor in the blend of P3HT: PCBM with PCBM as the acceptor [18,20,21]. Han et al. have reported highly oriented nanofibrils of such semiconducting materials by blading deposition in a microfluidic dragging manner [22,23]. Recently, Ding et al. reported a regular P3HT grating microstructure by using nanoimprinting lithography for studying the growing behavior of muscle cells [24]. Lin et al. proposed stripe-like structures by evaporative self-assembly of P3HT in a capillary tube [25]. 

In this study, we demonstrate a cost-effective method for fabricating polymeric micro patterns under flexible geometric confinement at a high temperature. Pure materials of PMMA and RR-P3HT are used. A flexible polydimethylsiloxane (PDMS) cover plate and a silicon wafer are used to establish a flexible geometric confinement. Then, the polymer solution is allowed to evaporate on a hotplate. With the time-elapsed evaporation, the solvent could be absorbed by the PDMS materials and the flat cover would become curved and bumped downwards, thus leading to a variable clearance for solvent evaporation. It is easier to remove the solvent than that of the parallel-plate confinement, because the entrapped solution for the latter can form denser vapor of the solvent and slow down the evaporation. In addition, the effect of gap height is also considered as an important influential factor for the formation of varied polymer microstructures. Finally, we demonstrate a simple field-effect transistor (FET) device using the RR-P3HT pattern as an active layer [26]. 

## 2. Materials and Methods 

### 2.1. Materials

PMMA (950 k) powder and RR-P3HT (87 k) powder were both purchased from Sigma-Aldrich (Shanghai, China). PMMA solutions with concentrations of 5–10% were obtained by resolving the PMMA powder in toluene, and the mixture was stirred for at least 10 h in fume hood. The RR-P3HT solutions with concentrations of 1–3 wt % were obtained by mixing 0.05 g powder into chlorobenzene after vigorously stirring under ambient condition for 1 h in fume hood. 

### 2.2. Experimental Process

**Figure 1:**
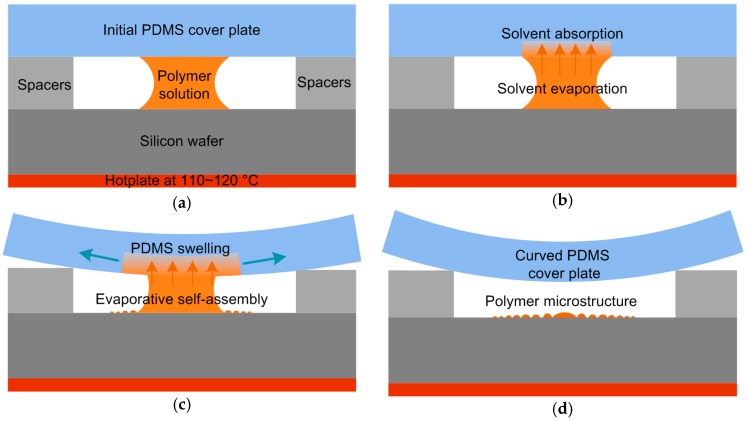
Schematic illustration of evaporating polymer solution in a flexible geometric confinement on a heated silicon wafer in the (**a**) initial state, (**b**) solvent absorption, (**c**) PDMS swelling, and (**d**) eventually curved state of the PDMS cover plate.

A thoroughly cleaned silicon wafer and glass-slides as substrate was placed on a hotplate. Two smaller pieces of 250 to 1000 μm-thick metal foils were used as spacers. As for the flexible geometric confinement, a PDMS plate 2 mm in thickness was placed over on the spacers to form a bridge and clearance (Figure 1). As for the rigid geometric confinement in comparison, a glass slide as a cover plate, was put onto the substrate to form a wedge-shape clearance (Figure S1c). After that, a drop of polymer solution with certain volume was supplied to the clearance, and allowed to evaporate. The temperature was adjusted to 110 °C for PMMA solution, while it was 120 °C for RR-P3HT solution. Once the polymer solutions were totally evaporated, microstructures could be observed on the substrate. In order to make it smoother, we further thermally annealed the as-prepared PMMA patterns on a 200 °C hotplate for half an hour. For comparison, the RR-P3HT was evaporated in both ambient and nitrogen atmosphere, and then the two samples were thermally annealed at 140 °C under nitrogen atmosphere for one hour.

### 2.3. FET Device Fabrication Based on RR-P3HT

Interdigital electrodes were fabricated on a thermally oxidized high-doped silicon wafer using ultraviolet (UV)-lithography, magnetic sputtering (of titanium and platinum in 100 nm thickness) and lift-off process (to remove the photoresist and the extra metal). After that, RR-P3HT patterns were fabricated over the interdigital electrodes using the proposed flexible geometric confined evaporative self-assembly approach. Finally, the simple FET device based on the RR-P3HT pattern was demonstrated.

### 2.4. Characterization

The morphologies of the polymer microstructures were characterized by field emission scanning electron microscope (FESEM), laser scanning confocal microscope (LSCM), and optical microscope. The nanostructure of the RR-P3HT were characterized by atomic force microscope (AFM, BRUKER, Billerica, MA, USA) using tapping mode, the scanning range was 2 × 2 μm and 5 × 5 μm, with a frequency of 0.7 Hz and 512 scanning lines per square. The absorbance spectra of the RR-P3HT films on glass slide were characterized using UV–vis Spectrometer (UV-3600, Shimadzu, Kyoto, Japan), with wavelength range of 310–800 nm. The electric test of the FET device was performed using a semiconductor analyzer (Keysight Technologies, Santa Rosa, CA, USA). 

## 3. Results and Discussion

### 3.1. Formation of the Flexible Geometric Confinement

Figure 1 illustrates the flexible cover plate deformation and thereafter the formation of curve-to-flat shape geometric confinement gradually as soon as the polymer solution was absorbed by PDMS. In the initial state of the cover-substrate set-up, the polymer solution did not evaporate fast because of the limited clearance. PDMS is a good absorber for the organic solvent, thus leading to an increment of the concentration of the initial polymer solution. Therefore, the PDMS cover would become curved gradually with time elapsed evaporation, and the clearance became larger at the outside region and smaller in the center region. By this means, the polymer solution could have a large evaporation rate at the outmost side, and retreat slowly with solvent evaporation. It would take a short time to form a curved shape of PDMS cover plate (Figure S2). We could consider the process of solvent absorption and PDMS swelling as a simple manner to make a curve-to-flat shape geometric confinement as reported Lin et al. [10,12]. Meanwhile, a high temperature near the boiling point of solvent would enhance the evaporative rate, thus leading to faster polymer deposition. 

### 3.2. Effect of Evaporative Openning on the Patterning of PMMA Microstructure

**Figure 2:**
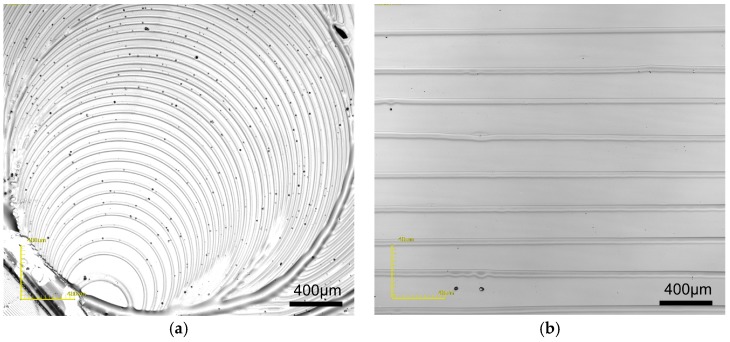
Patterns of PMMA obtained by evaporating (**a**) a single droplet in the center of the clearance and (**b**) wedge meniscus of PMMA solution on silicon wafer at 110 °C hotplate.

Figure 2a shows the circular rings of PMMA resulted from evaporating a single droplet of toluene solution in the center of the clearance, where the confinement was narrowed down (see also Figure S1a). In comparison, Figure 2b shows comparable regular stripes generated by evaporative-assembly from a meniscus on the vertical wedge-shape confinement which had a very larger open confinement (see also Figure S1b). 

**Figure 3:**
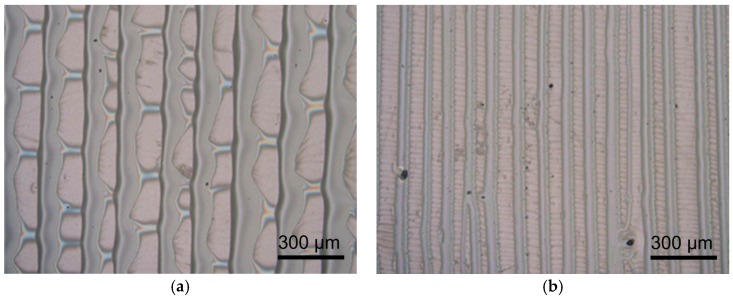
PMMA patterns obtained by evaporative self-assembly at varied gap heights of clearance: (**a**) 1000 μm and (**b**) 250 μm.

When the PMMA solution was trapped in a two parallel plates, the formation would be different. By adjusting the height of the evaporative opening, the widths and periodicities of the PMMA patterns could be both changed (Figure 3 and Figure S2). The evaporation rate would be increased with increasing the gap height of the clearance of evaporative opening. The relationship between the gap height and structure dimension is shown in Table 1. The width of the PMMA microstructure would increase with larger gap heights of clearance. Meanwhile, the density of the PMMA microstructure would be reduced with a higher gap of clearance for evaporation. 

**Figure 4:**
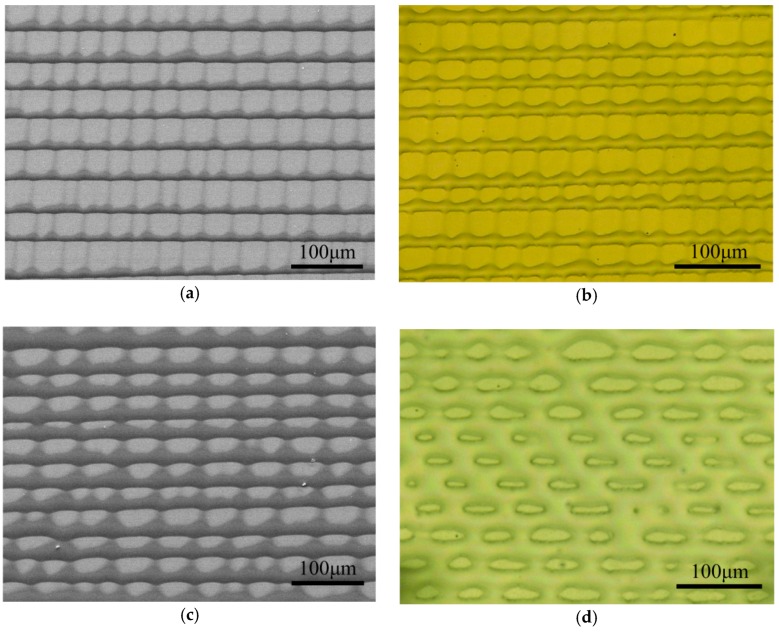
Morphologies of the PMMA microstructures. (**a**,**c**) SEM images of the as-prepared PMMA patterns obtained at varied gap heights of clearance; (**b**,**d**) optical microscope images of PMMA patterns after 200 °C annealing corresponding to (**a**,**c**), respectively.

Figure 4 demonstrates the obtained stripe patterns with high ordered crater-like structure by gelation under high temperature assisted evaporation. We can see that the microstripes have ordered distribution at both the lateral direction and the vertical direction. After being annealed on 200 °C hotplate, the PMMA patterns turn round shape at the edge due to the reflow under surface tension. Therefore, the PMMA patterns become shorter in height and larger in width (Figure 4b,d). At the same time, the wave-shaped patterns in the vertical direction become not as apparent as the patterns that without annealing. 

The mechanism for the facial formation of the polymer microstructure by the evaporative manner has been reported by many researchers [1,2,27,28,29]. The formation of PMMA at the vertical direction should be attributed to the coffee-ring effect. Meanwhile, the formation at the lateral direction should be due to the Marangoni effect under confined evaporation [30,31]. High temperature would lead to difference of surface tension at the liquid–air interface, and therefore drive the reflow of PMMA solution and the reshape of patterned stripes during evaporation. The stick-slip motion of the evaporating front could be helpful for the generation of the polymer patterns. There are several advantages for using high temperature to promote evaporative self-assembly formation. One of the greatest advantages is the enhancement of generation of the coffee-ring patterns under high evaporation temperature. On the other hand, a complex structure would be also obtained due to the Marangoni effect at high temperature-driven interfacial tension. The flexible geometric confinement would favor patterning polymer microstructures with various periodicities and widths.

### 3.3. Patterning of RR-P3HT Microstructures

**Figure 5:**
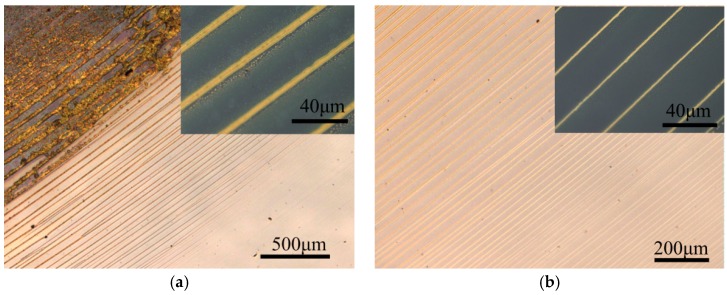
Optical microscope images of RR-P3HT patterns formed by evaporation under flexible geometric confinement, in the central region (**a**) and outside region (**b**).

Figure 5 shows the morphologies of the RR-P3HT microstructures. The widths of RR-P3HT stripes are thicker in the central region than that at the outside region due to the varied clearance of the geometric confinement. There are many casually distributed dense microdots surrounding the straight stripes with larger width obtained at the higher concentration region (Figure 5a). In contrast, the region between the finer stripes formed at low-concentration appears much cleaner (Figure 5b). 

**Figure 6:**
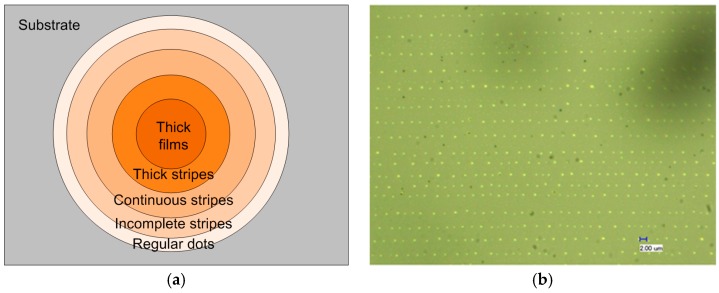

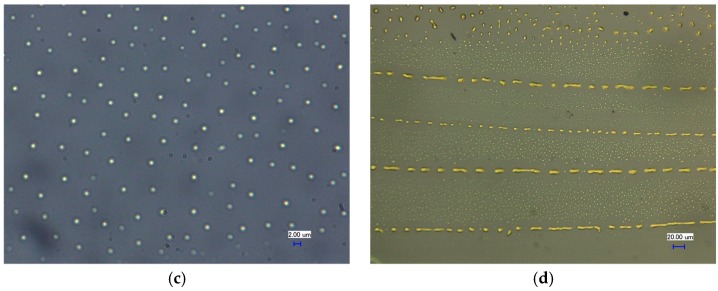
(**a**) Diagram depicting the various morphologies of RR-P3HT patterns formed by evaporative self-assembly under flexible geometric confinement. Optical microscope images of RR-P3HT patterns formed by evaporation under flexible geometric confinement: (**b**) regular dots, (**c**) irregularly distributed dots in between the stripes as seen in (**d**) incomplete stripes.

The concentration is the highest in the innermost region, and thus leading to a stacked thick film of RR-P3HT. Figure 6 shows the obtained RR-P3HT microstructures with various morphologies at different area (Figure S4). Figure 6a depicts the whole view of the obtained patterns of RR-P3HT microstructures with circular loops of various morphologies. The periodicity and width both becomes smaller ranging from the central region to the outmost region (Figure 6b). There are some small regions with regular stripes, while a dust would change the morphology of the stripe formation (Figure S4b). In the region not far away from the center, the widths become finer with few dots around the stripes (Figure S4c). At the outmost region where the evaporation occurred first, we can find some dotted patterns with regular distribution in both the vertical and lateral directions. In a certain region near the middle, we also find some incomplete stripes with casually distributed dilute microdots between the stripes (Figure 6c,d). Table 2 also lists the varied periodicities and widths at different distances from the interior region.

Different from the results reported by Lin et al. [1,2,12], the microstructure has various morphologies with increasing periodicities and widths simultaneously from the outmost to the center. The generation of dot-patterns should be due to the low concentration at the beginning. With solvent evaporating, the concentration at the three-phase contact line would become higher, so that the accumulation of the polymeric molecules would be increased, thus leading to a larger width. The main mechanism for formation of different morphologies in different regions can be depicted as below. The regularly distributed microdots were formed by gelation as part of the pinning-triple contact line in a rather low concentration. In contrast, the irregularly distributed microdots should be attributed to locally sputtering during receding of the evaporating front under the thermal effect. The surface tension driven Marangoni effect may also lead to the formation of the microdots by thermal re-flow [30,31]. 

**Figure 7:**
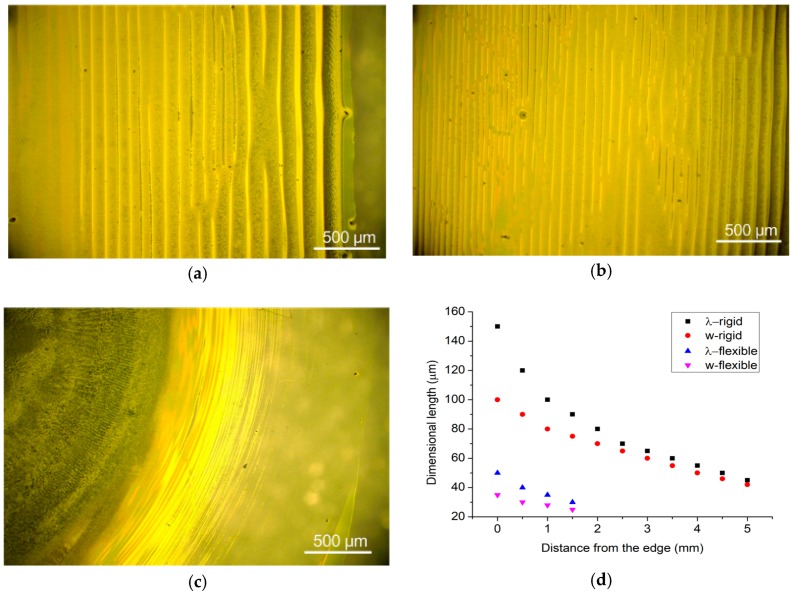
Optical microscope images of RR-P3HT obtained by evaporative self-assembly under rigid confinement (**a**,**b**) and flexible confinement (**c**) with a high solution concentration of 3 wt %. A thicker stripe pattern was obtained (**a**) near the edge and (**b**) distant from the edge; (**c**) very thick film surrounded by finer stripe patterns. (**d**) Comparison of periodicities (λ) and widths (w) of RR-P3HT stripe patterns resulted from the rigid and flexible cover plate.

We also investigate the self-assembly of RR-P3HT with higher concentration solutions. Figure 7 compares the RR-P3HT patterns that resulted from the proposed flexible geometric confinement with that from rigid wedge-shape geometric confinement. For the former, straight dense gradient stripes were obtained (Figure 7a,b). For the latter, thick films would be found in the middle region, while finer dense gradient stripes surround the thick film (Figure 7c). The variation of periodicities and widths for RR-P3HT patterns was also demonstrated in Figure 7d. In comparison with the rigid glass-slide cover plate, the flexible PDMS cover plate would form a curve shape via solvent swelling which would attract most of the solution to the middle part, leaving a small amount for the surrounding area, thus leading to a shorter range of stripe pattern.

**Figure 8:**
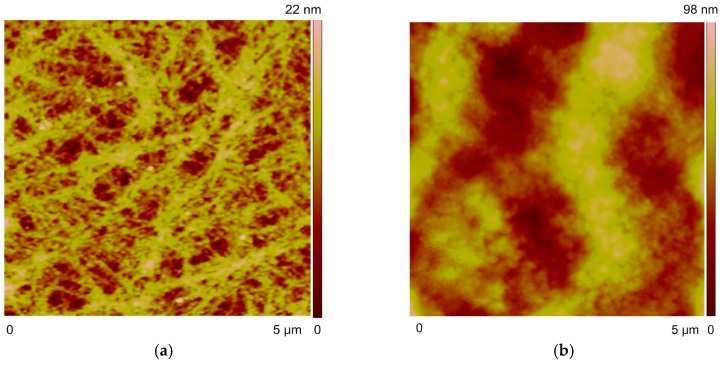

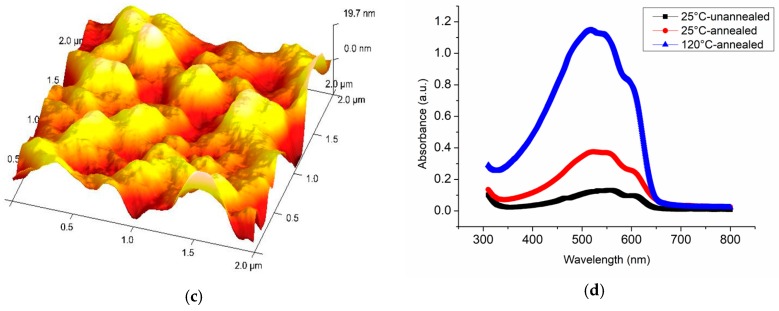
Characterization of nanostructure of RR-P3HT patterns. Atomic force microscope (AFM) images of the obtained RR-P3HT patterns: (**a**) nanofibrils observed on the stripes obtained by evaporative self-assembly at 120 °C and thermal annealing at 140 °C under nitrogen atmosphere; (**b**) nanochains observed in between two stripes of (**a**); (**c**) random distributed microstructure obtained at ambient condition at 25 °C without thermal annealing. (**d**) Absorbance spectra of the RR-P3HT patterns before and after thermal annealing, depicting the blue shift with thermal annealing.

Nanostructure can be also characterized with atom force microscope (AFM), depicting that the RR-P3HT microstructure would generate nanocrystals in forms of nanofibrils (Figure 8a) or nanochains with alignment orientation to the microstripe (Figure 8b) after thermal annealing. Meanwhile, the absorbance spectra also show a blue shift of the peak value (Figure 8d), indicating the formation of larger amount of nanocrystals. In contrast, the nanostructure in the unannealed RR-P3HT patterns are random distributed and less crystallized due to the fast evaporation (Figure 8c).

**Figure 9:**
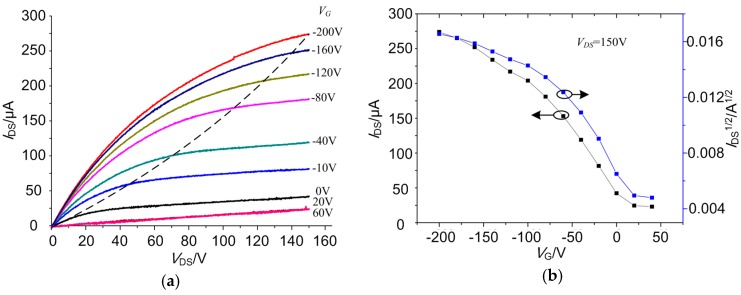
Performance of the RR-P3HT-based FET device: (**a**) output characteristic curve and (**b**) transfer characteristic curve.

The resultant RR-P3HT patterns can be used as an organic thin film transistor structure [5]. Interdigital electrodes were fabricated on a thermal-oxidized highly-doped p-type silicon wafer by using photolithography and lift-off methods. The silicon wafer was considered as a gate as well as a substrate. Meanwhile, the 500 nm-thick SiO_2_ layer was used as dielectric, with the 100 nm-thick platinum interdigital electrodes as both source and drain. Figure S5a demonstrates the patterned RR-P3HT on digital electrodes. Figure 9 shows the performance of the FET device fabricated with the RR-P3HT stripe patterns as an active layer, using the bottom electrode mode (Figure S5b). 

By simple estimation from both the output and transfer characteristic curves, we could obtain a carrier mobility of about 0.01 cm^2^/Vs, with threshold voltage of about 25 V, and the on/off current ratio of no larger than 10^2^ (Table 3). However, the above device performance is far from being applicable. The reason for the deterioration performance of RR-P3HT microstructures should be attributed to the high temperature which would render oxidation of the P3HT molecules. This should be the very reason for limited application of such vulnerable functional polymers like RR-P3HT in the form of microstructure patterning. Moreover, the large dielectric thickness of the FET device would lead to a rather high threshold voltage and working voltages. Better results are believed to be obtained if more RR-P3HT molecules were generated into finer crystalline by slower evaporation and thermal annealing in protective atmosphere [22,32,33]. For example, the whole experimental set-up could be carried out in a vacuum chamber while being performed at a high temperature. Moreover, better polymer crystalline would be generated by using a directional growth method like blade-deposition at a low temperature [20,34]. Nevertheless, we believe that many other polymers can be also patterned by employing the evaporative self-assembly manner using such a flexible geometric confinement.

## 4. Conclusions

In summary, we propose a flexible geometric confinement for evaporative self-assembly with variable clearance to form stripe patterns of structural and functional polymeric materials. PMMA can be formed into microstructures—such as circular rings, straight stripes, and hierarchical stripes—with various periodicities and widths under different height of clearance for evaporative assembly. Thermally enhanced evaporation would result in formation of complex structures under flexible geometric confinement. Moreover, a semiconducting type polymer RR-P3HT can be patterned into gradient microstructures, with various morphologies of thick film, stripes, discontinuous stripes, regularly-distributed dots, etc. RR-P3HT nanocrystals and polymer alignment could be found upon thermal annealing at 140 °C under nitrogen atmosphere. Finally, a simple FET device was demonstrated with the RR-P3HT patterns as functional layer on interdigital thin-film electrodes. Although it would be a simple method for generating a curve-to-flat confinement as well as improving the formation of gradient polymer microstructures, there are still limitations to the proposed flexible geometric confinement. For instance, the swelling of the PDMS cover plate would be a non-linear process during the absorption and deformation. The thermal control over the evaporative self-assembly process could not be so accurately. Moreover, the reproducibility seems not so good for such a flexible confinement by means of thermally enhanced evaporation and solvent swelling. Nonetheless, the evaporative self-assembly under flexible geometric confinement could pave the way for patterning many other functional materials for practical applications. 

## Figures and Tables

**Table 1:** Dimensional change of patterned PMMA microstructure with the gap height of clearance.

Gap Height/μm	Vertical Periodicity/μm	Vertical Width/μm	Lateral Periodicity/μm	Lateral Width/μm
250	80	35	25	3
500	100	45	120	20
1000	250	125	300	60

**Table 2:** Widths and periodicities of RR-P3HT microstructure distant from the central region using solution of low concentration of 1 wt %.

Distance/mm	Width/μm	Periodicity/μm
1	6	60
2	2.5	42
3	1	18
4	0.6	9

**Table 3:** Performance of the FET device with the RR-P3HT micropattern as an active layer.

*μ* (cm^2^/Vs)	*V*_th_ (V)	*I*_on_/*I*_off_
~0.01	~25	<10^2^

## Supplementary Materials

The following are available online at http://www.mdpi.com/2072-666X/9/3/124/s1.
