# Supplementary material for: Microstructure Formation of Functional Polymers by Evaporative Self-Assembly under Flexible Geometric Confinement
Source: Micromachines (Basel). 2018 Mar 12;9(3):124. doi: 10.3390/mi9030124 (PMC6187559; doi:10.3390/mi9030124)
Supplement: Supplementary file 1 [file micromachines-09-00124-s001.docx]

Supplementary Materials

Microstructure Formation of Functional Polymers by Evaporative Self-Assembly under Flexible Geometric Confinement

Xiangmeng Li ^1, 2^*, Xijing Zhu ^1, 2^ and Huifen Wei ^3^

^1^ Shanxi Province Key Laboratory of Advanced Manufacturing Technology, North University of China, Taiyuan, Shanxi, 030051, CHINA; xmli123@nuc.edu.cn , [zxj161501@nuc.edu.cn](mailto:zxj161501@nuc.edu.cn)

^2^ Institute of Precision & Special Manufacturing, School of Mechanical Engineering, North University of China, Taiyuan, Shanxi, 030051, CHINA; [xmli123@nuc.edu.cn](mailto:xmli123@nuc.edu.cn), [zxj161501@nuc.edu.cn](mailto:zxj161501@nuc.edu.cn)

^3^ Academy of Science and Technology, North University of China, Taiyuan, Shanxi, 030051, CHINA; whf_nuc@163.com

***** Correspondence: xmli123@nuc.edu.cn; Tel.: +86-0351-3921355

CONTENT:

Figure S1-S5


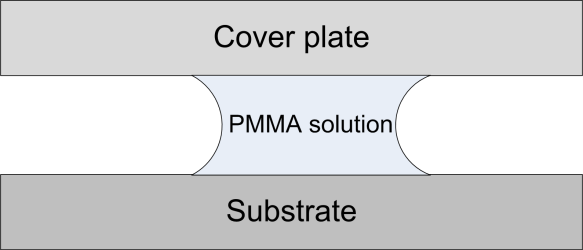

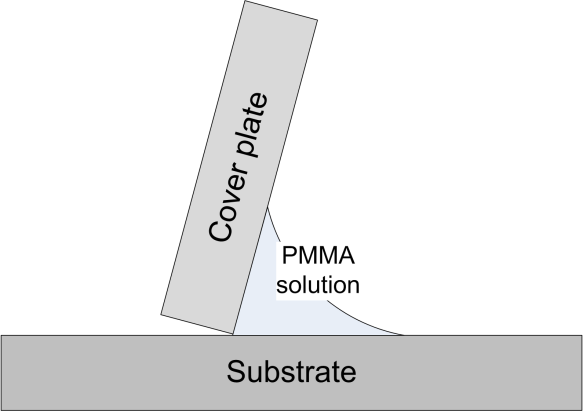


1. (b)


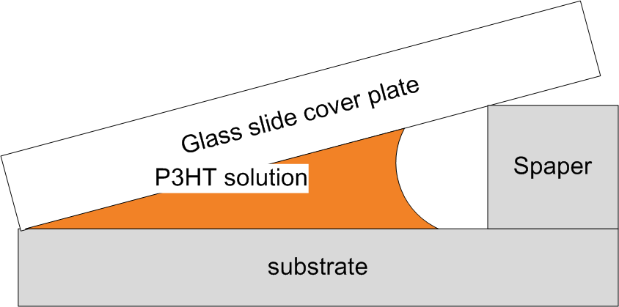


(c)

Figure S1. Diagram illustration of (a) evaporative self-assembly of PMMA toluene solution under two-parallel-plate confinement, (b) a large opening vertical wedge-shape confinement and (c) a rigid wedge-shape geometric confinement for RR-P3HT patterning.


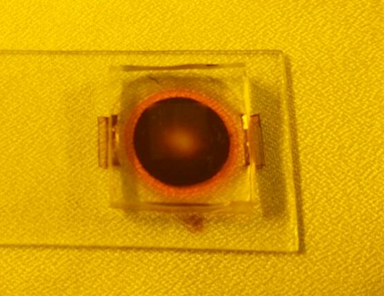

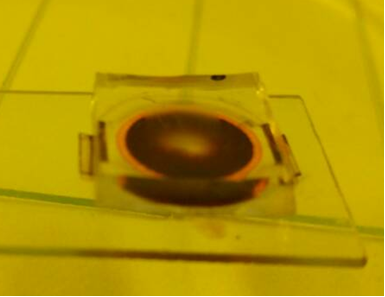


1. (b)

Figure S2. Photographs of the flexible geometric confinement set-up with PDMS cover plate on glass slide: (a) top view and (b) side view indicating the curved PDMS plate during solvent swelling.


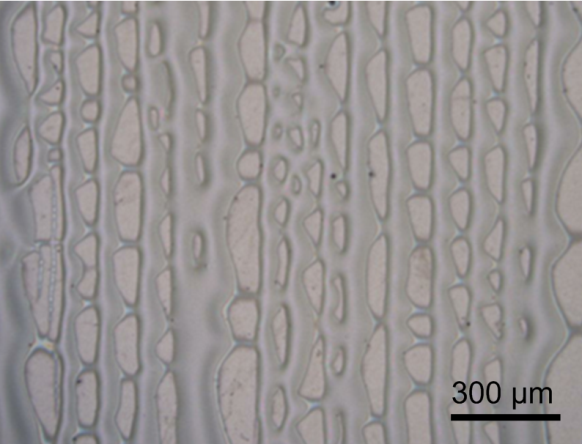


Figure S3. Laser confocal scanning microscope image of PMMA microstructure obtained by evaporative self-assembly under flexible geometric confinement, at a gap height of 500 μm.


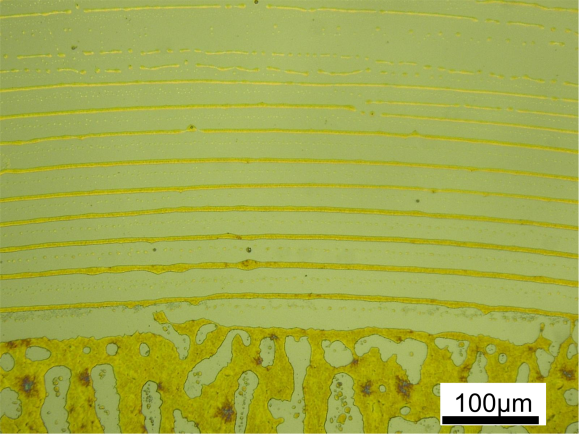

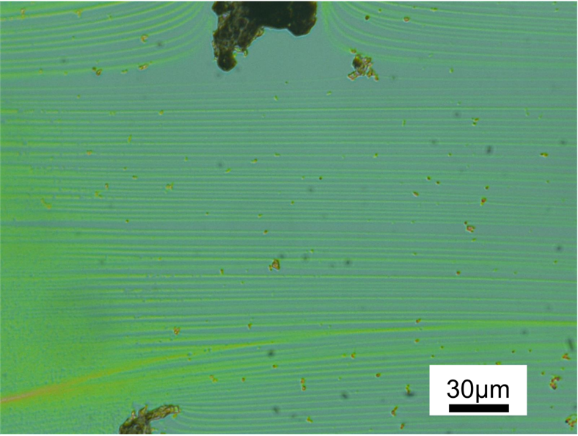


1. (b)


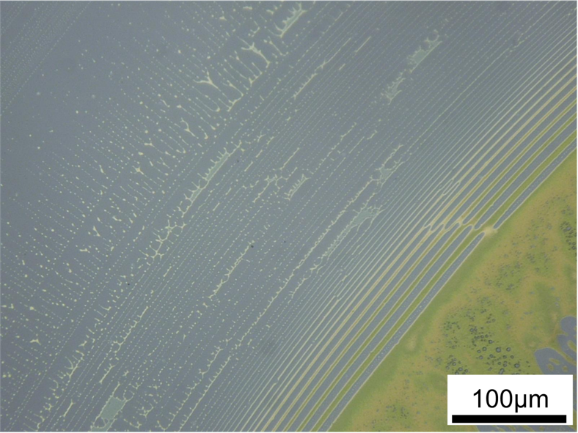


(c)

Figure S4. Optical microscope images of the various RR-P3HT microstructures.


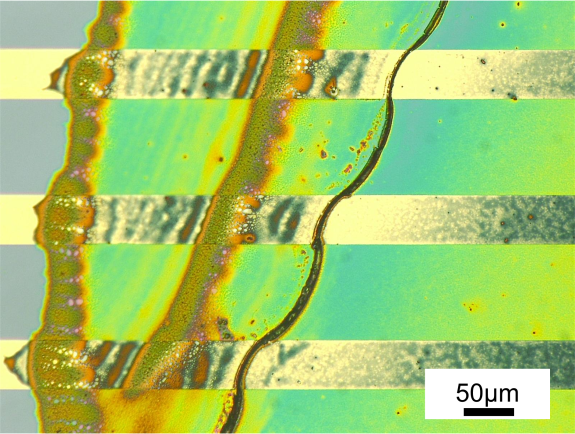

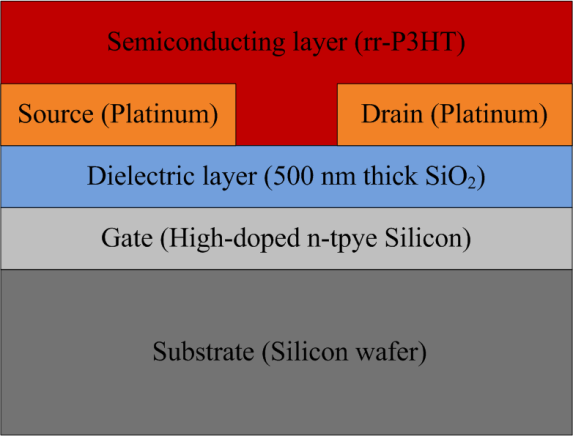


(a) (b)

Figure S5. (a) Optical microscope image of RR-P3HT patterns formed on the interdigital electrodes. (b) Schematic illustration of the field-effect transistor device.
